# Are parental physical activity and social support associated with adolescents’ meeting physical activity recommendations?

**DOI:** 10.11606/s1518-8787.2023057004362

**Published:** 2023-04-27

**Authors:** Edina Maria de Camargo, José Francisco López-Gil, Thiago Silva Piola, Jorge Mota, Wagner de Campos

**Affiliations:** I Universidade Federal do Paraná Centro de Estudos em Atividade Física e Saúde Programa de Pós-graduação em Educação Física Curitiba PR Brasil Universidade Federal do Paraná. Centro de Estudos em Atividade Física e Saúde. Programa de Pós-graduação em Educação Física. Curitiba, PR, Brasil; II Universidad de Castilla-La Mancha Health and Social Research Center Cuenca Spain Universidad de Castilla-La Mancha. Health and Social Research Center. Cuenca, Spain; III Harvard University Harvard T.H. Chan School of Public Health Department of Environmental Health Boston MA USA Harvard University. Harvard T.H. Chan School of Public Health. Department of Environmental Health. Boston, MA, USA; IV Universidad de Las Américas One Health Research Group Quito Ecuador Universidad de Las Américas. One Health Research Group. Quito, Ecuador; V Universidade do Porto Faculdade de Desporto Centro de Investigação em Actividade Física, Saúde e Lazer Porto Portugal Universidade do Porto. Faculdade de Desporto. Centro de Investigação em Actividade Física, Saúde e Lazer. Porto, Portugal

**Keywords:** Adolescent, Parent-Child Relations, Sedentary Behavior, Exercise, Sedentary Behavior, Social Support

## Abstract

**OBJECTIVE:**

To verify whether parental physical activity and social support are associated with adolescents meeting physical activity recommendations.

**METHODS:**

This is a cross-sectional study that selected 1,390 adolescents (59.6% girls) from Curitiba, Paraná, Brazil. The IPAQ (International Physical Activity Questionnaire), QAFA (Physical Activity Questionnaire for Adolescents), and ASAFA (Social Support for Physical Activity Practice in Adolescents) questionnaires were applied. Binary logistic regression was used to test the relationship among the study variables.

**RESULTS:**

For boys, having parents who “always attend” (OR = 1.96; 95%CI: 1.16–3.32) and having parents or legal guardians who meet the PA recommendations (OR = 2.78; 95%CI: 1.76–4.38) were associated with meeting the PA recommendations. Odds were greater after adjusting for socioeconomic status (OR = 3.47; 95%CI: 1.73–6.96) and schooling level (OR = 4.20; 95%CI: 1.96–9.02). For girls, those with parents or legal guardians who “sometimes encourage them” (OR = 0.61; 95%CI: 0.37–0.98) had lower odds of meeting PA recommendations. These odds were higher after adjusting for socioeconomic status (OR = 2.11; 95%CI: 1.36–3.29) and schooling level (OR = 4.30; 95%CI: 2.41–7.69).

**CONCLUSIONS:**

Boys and girls were more likely to meet PA recommendations daily by having parents who meet PA recommendations than by receiving parental social support. These results could help establish future interventions aimed at modifying behaviors related to PA in adolescents.

## INTRODUCTION

The increase in the prevalence of insufficient physical activity (PA) (i.e., not meeting the daily recommendations), both in developed and developing countries, is of great concern^1,2^. The largest worldwide study on PA in children and adolescents, published by Guthold et al. (2020), analyzed the evolution from 2001 to 2016 of 1.6 million young students in almost 300 school-based surveys in 146 countries and territories^1^. Eight out of ten children and adolescents aged 11 to 17 years did not meet the PA recommendations^1^. The data also show no significant improvement in these levels worldwide in the last 15 years^1^. Therefore, if this trend continues, the World Health Organization (WHO) will not achieve their goal of a 15% reduction in physical inactivity in the population by 2030^3^.

According to the WHO recommendations, adolescents (aged 13 to 18 years) should perform a minimum of 60 minutes/day of moderate-to-vigorous physical activity (MVPA) to benefit their health^4^. However, for those who do not engage in any PA, some activity is better than nothing^4^. For those who engage in PA but insufficiently, increased activity will help them meet the recommendations. For those who already meet the PA recommendations, further activity will provide even more benefits^4^.

In Brazil, 54.3% of Brazilian adolescents do not meet the PA recommendations^5^. According to Guthold et al.^1^, in a global study, 84% of Brazilian adolescents were insufficiently active (i.e., did not meet the recommended minimum PA for health benefits)^4^. Considering sex, girls were more insufficiently active (89%) than boys (78%)^1^. Several factors could be associated with these results, including sociodemographic, economic, or social/family support indicators, among others^6^.

Perceived social support (SS) (e.g., encouraging, practicing together, providing transportation, attending, and praising PA practice) in adolescents is associated with higher PA and, therefore, with higher odds of meeting the PA recommendations^6^. Accordingly, several national and international studies have indicated that the level of SS from family as well as from peers is related to higher levels of PA in adolescents^6^. Moreover, parents practicing PA are an advantageous role model for children to become involved in PA^8,9^.

Although studies have investigated the association between parents’ or legal guardians’ PA levels and SS in isolation, few have examined these variables together^8,9^. This study aims to analyze the association between adolescents meeting PA recommendations and the PA levels of parents or legal guardians. It also investigates whether the SS received from parents or legal guardians to practice PA is associated with meeting PA recommendations among adolescents. Our results could increase the understanding of how to improve MVPA levels among adolescents by SS and role models such as parents or legal guardians.

This study is relevant since reducing physical inactivity is a serious concern to worldwide population health^3^. Moreover, family SS and family role models are seemingly significant in PA behavior^6^. Data from low- to middle-income countries are essential to understand how these socioeconomic variables could increase PA and, therefore, to establish effective public policies considering each context.

This study thus sought to verify whether parental PA and social support are associated with adolescents meeting physical activity recommendations.

## METHODS

The study followed the standards of research involving human beings according to resolution number 466/2012 of the National Health Council and was approved by the Research Ethics Committee of the *Universidade Federal do Paraná* (CAAE: 98133218.8.0000.0102). All participants provided written informed consent (signed by parents or legal guardians).

This was a cross-sectional study conducted in 2018 with a representative sample of adolescents aged 15 to 17 years. All participants were enrolled in public secondary schools.

For the preliminary sample size calculation, an association of odds ratio (OR = 1.49) was considered between SS and PA^11^, with a 50% prevalence of being insufficiently active ^12^ and a confidence level of 95% (α = 0.05) with a power of 80% (β = 0.20), resulting in a minimum sample of 804 subjects. However, a 30% increase was considered for possible losses and refusals, requiring a minimum sample of 965 subjects.

To select the study sample, multistage cluster sampling was conducted in three stages: First stage – all public schools were stratified according to each of the nine administrative regions of the city of Curitiba; Second stage – two schools were selected from each of the nine regions; Third stage – from these 18 selected schools, a simple random selection of a school class was made for each school year according to the number of students required for each region (for both sexes).

All students in each class were invited to participate in the study (2,506 adolescents). Adolescents who did not present the informed consent form (n = 104), who refused to participate in the study or who were absent on the day of collection (n = 56) were not included in the study. Furthermore, adolescents who presented physical and/or cognitive limitations (informed by the school) (n = 12) and those who were 18 years old (i.e., adults) were excluded (n = 125). Moreover, those who answered the questionnaires incorrectly or did not answer regarding their PA level (n = 819) were considered missing data. Finally, the analytical sample of the study included 1390 adolescents.

However, preliminary statistical analyses showed that considering 80% power (β = 20%), a 95% confidence level (α = 5%), and a 34% prevalence of adolescents with low SS who did not meet the PA recommendations allowed the identification of statistically significant odds ratios above OR = 1.36 and below OR = 0.73.

### Physical Activity – Parents or Legal Guardians and Adolescents

The MVPA level of parents or legal guardians was measured using the IPAQ (International Physical Activity Questionnaire) – short version, in which parents or legal guardians reported “how many days a week” and “how long a day” they practiced MVPA during a normal week^13^. The time dedicated to each PA practice (leisure and transportation) per week was categorized according to the current PA recommendations (“meeting PA recommendations”: ≥ 150 minutes per week of MVPA practice and “not meeting PA recommendations”: < 150 minutes per week of MVPA practice)^4^.

The PA level of adolescents was assessed by the *Physical Activity Questionnaire for Adolescents* (QAFA)^14^. Sallis et al.^15^ first developed this questionnaire in the checklist format for North American adolescents and Farias-Júnior et al.^14^ translated and adapted it for Brazilian adolescents. The questionnaire is a list of 24 types of MVPAs and allows adolescents to add other activities. In Brazilian adolescents, the questionnaire showed good reproducibility (ICC = 0.88; 95%CI: 0.84–0.91) and concurrent validity compared to 24-hour recall (r = 0.62; p < 0.001)^14^. Participants’ PA level was divided into “meeting PA recommendations” (≥ 60 minutes per day of MVPA) and “not meeting PA recommendations” (< 60 minutes per day of MVPA practice). This classification followed the WHO recommendation that adolescents (13 to 18 years old) should perform at least 60 minutes/day of MVPA to obtain health benefits^4^.

### Social Support from parents or Legal Guardians for Physical Activity Practice

The social support (SS) parents or legal guardians offer to children when practicing PA was measured by the 5-item scale *Social Support for Physical Activity Practice in Adolescents* (ASAFA), which has satisfactory consistency (parental SS α ≥ 0.77 and CRI ≥ 0.83)^16^. Adolescents reported the frequency (never = 1, rarely = 2, frequently = 3, always = 4) with which parents offered some type of SS for PA practice (e.g., encouragement, practice, transportation, attendance, comment) during a typical or normal week^16^ for the question *“How often do your parents encourage you to practice PA? Do they practice PA with you? Do they take you or provide transportation to the place where you practice PA? Do they attend you in PA? Do they comment about your PA practice?”*. To compare this work with similar studies^17^, the response options “rarely” and “frequently” were combined and classified as “sometimes”. The weekly frequency categories of SS were thus classified as “never”, “sometimes”, and “always”.

### Sociodemographic Information and Health

Sex and age were self-reported. Body weight was measured by a trained researcher using a portable digital anthropometric scale (W721, Wiso, Brazil) with a 100 g resolution and 150 kg capacity. The adolescents were barefoot, wearing only light clothing, and guided to maintain their anatomical position. They were told to stand with their backs turned to the scale and to distribute their body mass equally between both feet. A portable stadiometer (W721, Wiso, Brazil) was used to measure the total height of the participants (in cm). Participants were in the anatomical position with their head in Frankfurt plane and performed an inspiratory apnea at the time of measurement. Two measurements were made, and in case of divergences greater than 0.1 centimeters (cm) between measurements, the procedure was redone^18^. The technical error of measurement was obtained by several repeated measurements on the same subject performed by the same observer, taking the differences and entering them into an appropriate equation. For intraobserver TEM of the two measurements taken, the equation used was $\sqrt{\Sigma D/2N}$ , where “D” is the difference between measurements and “N” is the number of individuals measured^19^. The intraclass correlation coefficient (ICC) was also estimated to verify the reliability of the measurements, resulting in 0.96.

Body mass index (BMI) was estimated by dividing body weight (in kilograms) by height (in meters squared). BMI was then converted into Z scores following the WHO criteria for sex and age, as follows: “low weight” = < -2 SD (standard deviation), “normal weight” = ≥ -2 SD and < +1 SD, “overweight” = ≥ +1 SD and < +2 SD, and “obesity” = ≥ +2 SD, classified as “no excess weight” and “excess weight”^20^. The schooling level of the main family breadwinner and their socioeconomic status (SES) were evaluated with a standardized questionnaire^21^. Schooling level was classified as elementary school, secondary school, or higher levels. SES was classified into three categories: “low” (class C + D), “medium” (class B1 + B2), and “high” (class A1 + A2) (ABEP, 2016)^21^.

### Data Collection

Adolescents took home the questionnaire along with an informed consent form for their parents or legal guardians. Participants who had parental consent to participate in the study answered the questionnaire at school during physical education classes with the help of the research staff. All researchers who participated in data collection were previously trained in a pilot project.

### Data Analysis

After the Kolmogorov–Smirnov test was used to identify data distribution, descriptive statistics were used to distribute absolute and relative frequencies. The chi-square test (χ^2^) was used to verify differences in proportions regarding sex and recommended levels of MVPA in adolescents. Binary logistic regression analysis was used to test the relationship among the study variables (i.e., between the level of MVPA of parents or legal guardians and their SS for PA practice and the level of MVPA of adolescents). The value of p ≤ 0.20 was adopted for the entry of the variable in the adjusted model according to the enter criterion. The interaction between age, nutritional status, main breadwinner’s schooling level, and SES logistic regression analysis models was then introduced (multiplication of the possible moderating variable with the independent variable, e.g., age × SS). Twenty interactions were tested regarding age, nutritional status, main breadwinner’s schooling level, and SES with the SS scale and MVPA parents (Tables 2 and 3). Statistical significance was established at p < 0.05.

To avoid bias related to the complex sample selection process (stratified sampling), association analyses were corrected by the complex design using the SPSS software version 23.0 complex sample command. This procedure was used to ensure that the estimates reflected the population data of the elementary units in the sample.

## RESULTS

The sample included 1390 adolescents (59.6% girls) and their respective parents or legal guardians. Table 1 shows the study variables stratified by sex. Nutritional status (p = 0.001), SES (p = 0.015), and the level of MVPA of parents (p = 0.021) showed significant differences between sexes.

**Table 1:** Sociodemographic information and health and data on social support and physical activity, stratified by sex (n = 1,390).

Sociodemographic Information and Health	Boys		Girls		p	Total	
		
	n	%	n	%		n	%
Age							
15	175	37.7	289	62.3	0.090	464	33.4
16	203	40.5	298	59.5		501	36.0
17	184	43.3	241	56.7		425	30.6
BMI							
Not excess weight	469	38.8	741	61.2	0.001^c^	1210	87.1
Excess weight	93	51.7	87	48.3		180	12.9
The main breadwinner’s schooling level							
Elementary school	217	40.3	322	59.7		539	38.8
Secondary school	192	38.8	303	61.2	0.490	495	36.6
Higher schooling levels	153	43.0	203	57.0		356	25.6
SES							
Low SES	93^a^	35.8	167^a^	64.2		260	18.7
Medium SES	348^a^	40.1	520^a^	59.9	0.015^d^	868	62.4
High SES	121^a^	46.2	141^b^	53.8		262	18.8
**SS parents/Legal guardians^e^**	**n**	**%**	**n**	**%**	**p**	**n**	**%**
Encouragement							
Never	104	37.5	173	62.5		277	19.9
Sometimes	313	40.3	463	59.7	0.168	776	55.8
Always	145	43.0	192	57.0		337	24.2
Practice together							
Never	205	37.4	343	62.6		548	39.4
Sometimes	288	42.0	398	58.0	0.060	686	49.4
Always	69	44.2	87	55.8		156	11.2
Transportation							
Never	268	40.5	394	59.5		662	47.6
Sometimes	176	40.1	263	59.9	0.955	439	31.6
Always	118	40.8	171	59.2		289	20.8
Attendance							
Never	239	39.4	367	60.6		606	43.6
Sometimes	235	40.4	346	59.6	0.355	581	41.8
Always	88	43.3	115	56.7		203	14.6
Comment							
Never	209	38.7	331	61.3		540	38.8
Sometimes	232	43.9	296	56.1	0.992	528	38.0
Always	121	37.6	201	62.4		322	23.2
**Level of MVPA^f^**	**n**	**%**	**n**	**%**	**p**	**n**	
Parents/Legal Guardians							
Does not meet	411	42.5	557	57.5	0.021^c^	968	69.6
Meets (150 minutes/week)	151	35.8	271	64.2		422	30.4
Adolescents							
Does not meet	367	65.3	564	68.1	0.296	931	67.0
Meets (60 minutes/day)	195	34.7	264	31.9		459	33.0

SES: socioeconomic status; BMI: body mass index; SS: social support; MVPA: moderate-to-vigorous physical activity.

^a,b^ Each subscript letter indicates a subset of sex (male and female) categories whose column proportions do not differ significantly from each other, Bonferroni’s post hoc test;

^c^ Statistically significant for the chi-square test for continuity correction; ^d^ Statistically significant for the linear association by chi-square test; p < 0.05. ^e^ *SS Parents/Legal Guardians: How often do your parents encourage you to practice PA (physical activity)? Do they practice PA with you? Do they take you or provide transportation to the place where you practice PA? Do they attend you in PA? Do they comment about your PA practice?*

^f^ Meets or does not meet physical activity recommendations (WHO, 2010)^6^.

Boys had higher odds of meeting PA recommendations when parents “always attended them” for PA practice (OR = 1.96; 95%CI: 1.16–3.32). Similarly, boys had greater odds of meeting PA recommendations when their parents or legal guardians met the recommendations (OR = 2.78; 95%CI: 1.76–4.38). Boys were more likely to meet PA recommendations when their parents met the recommendations and had a higher SES than when parents did not meet the recommendations (OR = 3.47; 95%CI: 1.73–6.96). Boys whose parents met the PA recommendations and had a high schooling level were also more likely to meet recommendations than boys whose parents or legal guardians did not meet PA recommendations (OR = 4.20; 95%CI: 1.96–9.02) (Table 2).

**Table 2:** Association between parental social support and level of physical activity of parents/legal guardians and recommended MVPA levels in adolescent boys (n = 1,390).

SS parents/legal guardians^a^	Recommended levels of MVPA in adolescent boys (60 minutes/day)													

	Unadjusted					Adjusted^b^			Interaction^c^			Interaction^d^		
			
	n	%	OR	95%CI	p	OR	95%CI	p	OR	95%CI	p	OR	95%CI	p
Encouragement														
Never	104	37.5	1											
Sometimes	313	40.3	1.40	0.74–2.62	0.305									
Always	145	43.0	1.35	0.84–2.16	0.215									
Practice together														
Never	205	37.4	1											
Sometimes	288	42.0	0.82	0.40–1.66	0.577									
Always	69	44.2	0.85	0.45–1.60	0.613									
Transportation														
Never	268	40.5	1											
Sometimes	176	40.1	0.86	0.52–1.44	0.570									
Always	118	40.8	1.37	0.80–2.33	0.247									
Attendance														
Never	239	39.4	1			1								
Sometimes	235	40.4	1.26	0.66–2.41	0.484	1.28	0.71–2.30	0.418						
Always	88	43.3	1.90	1.07–3.39	0.029	1.96	1.16–3.32	0.012^e^						
Commente														
Never	209	38.7	1			1								
Sometimes	232	43.9	1.55	0.84–2.87	0.165	1.50	0.85–2.67	0.166						
Always	121	37.6	0.73	0.44–1.20	0.218	0.78	0.49–1.27	0.328						
MVPA Parents (150 minutes/week)														
Does not meet	411	42.5	1			1			1			1		
Meets	151	35.8	2,68	1.69–4.24	0.000	2.78	1.76–4.38	0.000^e^	4.20	1.96–9.02	0.000^e^	3.47	1.73–6.96	0.000^e^

SS: social support; MVPA: moderate-to-vigorous physical activity.

^a^ *SS parents/legal guardians: How often do your parents encourage you to practice PA (physical activity)? Do they practice PA with you? Do they take you or provide transportation to the place where you practice PA? Do they attend you in PA? Do they comment that you are doing your PA practice?*

^b^ Adjusted by the enter criterion, p ≤ 0.20 was adopted for the entry of the variable in the adjusted model.

^c^ By main breadwinner’s schooling level (high).

^d^ By socioeconomic status (high).

^e^ Statistically significant for the test; p < 0.05.

Girls had a lower odds of meeting PA recommendations when their parents “encouraged them sometimes” for PA practice (OR = 0.61; 95%CI: 0.37–0.98). In turn, they were more likely to meet PA recommendations when their parents met the PA recommendations (OR = 2.04; 95%CI: 1.46–2.87). Girls whose parents or legal guardians met PA recommendations and had a high SES were also more likely to meet the recommendations than those whose parents or legal guardians did not meet the PA recommendations and had lower SES (OR = 2.11, 95%CI: 1.36–3.29). Moreover, girls whose parents or legal guardians met the PA recommendations and had a high schooling level were more likely to meet the recommendations than those whose parents or legal guardians did not meet the PA recommendations (OR = 4.30, 95%CI: 2.41–7.69) (Table 3).

**Table 3:** Association between parental social support and level of physical activity of parents/legal guardians and recommended MVPA levels in adolescent girls (n = 1,390).

SS parents/legal guardians^a^	Recommended levels of mvpa in adolescent girls (60 minutes /day)													

	Unadjusted					Adjusted^b^			Interaction^c^			Interaction^d^		
			
	n	%	OR	95%CI	p	OR	95%CI	p	OR	95%CI	p	OR	95%CI	p
Encouragement														
Never	104	37.5	1			1								
Sometimes	313	40.3	0.65	0.38–1.08	0.097	0.61	0.37–0.98	0.042^e^						
Always	145	43.0	1.01	0.66–1.55	0.966	1.06	0.71–1.59	0.771						
Practice together														
Never	205	37.4	1											
Sometimes	288	42.0	1.33	0.74–2.38	0.883									
Always	69	44.2	1.39	0.80–2.40	0.685									
Transportation														
Never	268	40.5	1											
Sometimes	176	40.1	0.78	0.49–1.25	0.301									
Always	118	40.8	1.07	0.68–1.69	0.774									
Attendance														
Never	239	39.4	1											
Sometimes	235	40.4	0.97	0.55–1.68	0.892									
Always	88	43.3	1.08	0.65–1.80	0.758									
Comment														
Never	209	38.7	1			1								
Sometimes	232	43.9	1.36	0.84–2.20	0.209	1.25	0.82–1.89	0.302						
Always	121	37.6	1.41	0.91–2.18	0.127	1.46	0.96–2.23	0.075						
MVPA parents (150 minutes/week)														
Does not meet	411	42.5	1			1			1			1		
Meets	151	35.8	1,99	1.42–2.80	0.000	2.04	1.46–2.87	0.000^e^	4.30	2.41–7.69	0.000^e^	2.11	1.36–3.29	0.001^e^

SS: social support; MVPA: moderate-to-vigorous physical activity.

^a^ *SS parents/legal guardians: How often do your parents encourage you to practice PA (physical activity)? Do they practice PA with you? Do they take you or provide transportation to the place where you practice PA? Do they attend you in PA? Do they comment that you are doing your PA practice?*

^b^ Adjusted by the enter criterion, p ≤ 0.20 was adopted for the entry of the variable in the adjusted model.

^a^ By main breadwinner’s schooling level (high).

^d^ By socioeconomic status (high).

^e^ Statistically significant for the test; p < 0.05.

## DISCUSSION

Overall, this study’s results indicate differences in how perceived SS from parents or legal guardians is associated with PA practice. Some reasons could (at least partially) explain these results, including, parents’ schooling and socioeconomic level, adolescents’ age and gender, and the region of the country where the family lives. For boys, meeting PA recommendations was associated with having parents who “always attend them” in PA practice. For girls, meeting PA recommendations was inversely associated with having parents who “encourage them sometimes” for PA practice. Furthermore, having parents or legal guardians who met PA recommendations was associated with adolescents meeting PA recommendations. However, variables related to sample characteristics (i.e., parents’ schooling level and SES) could partially explain the results obtained.

Several studies have indicated that perceived SS from family and parents or legal guardians who meet PA recommendations are associated with higher levels of PA among adolescents^6^. In this study, for boys, parental support and having parents who met the PA recommendations were associated with meeting PA recommendations. In a systematic review of reviews, including studies that were rated with very high quality and relevance, evidence showed that parents are essential to promote PA during childhood and adolescence^22^. Similarly, Prado et al.^17^ found that boys who always received positive reinforcement from parents engaged in more PA. Other studies have also shown that among types of social support to PA, adolescents most reported “encouragement”^7,9,22^. Yao and Rhodes^9^ also found that parental encouragement was the SS most strongly related to their children’s PA. In this study, however, most of the SS types analyzed presented no significant association with PA practice among adolescents. This finding corroborates studies by Fermino et al.^11^, Prado et al.^17^, and Piola et al.^10^, all three carried out in Southern Brazil, as in this study. This is likely because family support can vary according to sample characteristics (e.g., parents’ schooling level, SES, PA level, region where adolescents live, built environment, opportunities for being active, etc.), which would partially explain the difference found between studies.

Parental SS is essential to engage adolescents in PA^23^; however, the scientific literature has focused more on social support from friends than from families among older adolescents^23^. Nevertheless, our results indicate that having active parents (i.e., parents who meet PA recommendations) as role models positively influences PA level among adolescents. Although adolescents may not recognize the relevance of the SS of parents, they recognize the importance their parents have as role models for PA practice^23^.

For girls, meeting PA recommendations was inversely associated with having parents or legal guardians who “encourage them sometimes” but related to having parents or legal guardians who met the PA recommendations. The study by Piola et al^10^ investigated social support with parents and friends of adolescents in the city of Curitiba, showing that adolescence is a period of transition and adolescents tend to listen more to their friends and less to their parents. The authors also found that parents “always” praising their children’s activities was associated with higher PA practice in both girls and boys (OR = 2.60; 95%CI: 1.01–6.71). Associations between different types and sources of SS can also vary according to the age and sex of adolescents^7^. Accordingly, Prado et al.^17^ found a greater practice of PA (e.g., 60 minutes of MVPA five or more days per week) when parents practiced PA together with their daughters. Parents and family members should therefore encourage girls to practice PA. This shows that an increased SS raises the level of PA in adolescents^7^.

A systematic review^24^of cross-sectional studies that examined both children’s and parents’ PA in countries with a high development index (United States, United Kingdom, Australia, Canada, France, and Portugal) found that fathers’ MVPA was associated with their children’s MVPA whereas children’s participation in vigorous activities was associated with fathers’ practice of vigorous PA^24^. For mothers, the practice of moderate PA was associated with their children’s moderate PA^24^. Both associations were found for weekdays and weekends^24^. Encouragement from parents or legal guardians is important for physically active children and adolescents to be more physically active in adulthood, considering that physically active adults are more likely to have an adequate health status^25^. This could reduce the odds of having health-related problems later in life and cause additional public health costs^28,29^.

The physical activities reported by parents in their childhood and adolescence were also associated with higher levels of PA among adolescents^25^. Adolescents with parents physically active in the past and in the present were six times (OR = 6.67; 95% CI: 1.94–2.79) more likely to be physically active than adolescents whose parents or legal guardians were not physically active in the past^25^. Understanding the parental constructions that measure PA since childhood is therefore essential^24^.

Adolescent girls whose parents meet the PA recommendations are almost twice as likely to meet the recommendations. Together, these findings show that parents’ regular practice of PA combined with parental support are important for adolescents’ PA and MVPA practice. Regarding the specific types of SS associated with PA, adolescents tend to have increased self-esteem when people close to them are present and provide feedback on their behavior, which could increase their perception of self-efficacy^8^. This could also help engage adolescents in higher levels of PA^8^. Encouragement from parents or legal guardians can directly affect the active participation of adolescents since it could help the latter group to establish daily routines that include PA.

These results confirm the importance of the family environment on adolescents’ PA levels^7,9,22^. This indicates that the family environment influences the dynamic behavior of adolescents by the attitudes of parents or legal guardians, such as offering support and, especially, being physically active^30^. In this study, both boys and girls were more likely to meet PA recommendations when having parents who met the PA recommendations than receiving parental SS. Nevertheless, further studies are needed to confirm parents’ influence on meeting PA recommendations in adolescents.

More studies must also investigate the interaction between parental schooling level and SES since this study showed that boys and girls are more likely to meet PA recommendations if the main family breadwinner has both a high schooling level and high SES. In this sense, parents with high schooling levels can better understand the benefits of PA and thus engage and encourage their children to engage in more activities. Considering the results of the Global Matrix report, a significantly low negative correlation was observed between overall PA and some sociodemographic indicators (e.g., Human Development Index and the gross national income per capita)^30^. Therefore, developing effective strategies to offer PA opportunities for all should be a national public health concern in all countries regardless of SES.

### Limitations

Some points should be considered when interpreting the results of this study. One of them is reverse causality, a common characteristic in studies with a cross-sectional design, which does not allow establishing cause-and-effect associations or determining the direction of the associations. Longitudinal studies are needed for a more robust confirmation of these associations. This study was developed in one Brazilian city that has the typical characteristics of well-developed urban centers, that is, the study findings cannot be applied to rural centers and other cities in the country.

The sample included only adolescent students from public schools and findings cannot apply to higher socioeconomic classes. However, the representative sample and statistical analyses ensure an interpretation of the data for large populations of public schools, relevant in the field of interventions related to public and preventive health.

The use of reported measures depends on the accuracy and recall power of the respondent’s responses. To reduce this bias, researchers were trained to attend adolescents in answering questionnaires. The instrument used to measure PA does not allow the identification of all domains and contexts of the practice of activities. Obtaining a more accurate measure would therefore reduce type 1 error. However, since this is a large study with a representative sample, using questionnaires proved to be the best alternative.

## CONCLUSION

In this study, adolescents with parents or legal guardians who met the PA recommendations were more likely to meet the PA recommendations. Some types of SS were associated with meeting PA recommendations among adolescents, with differences between sexes. Social support from parents or legal guardians and physically active role models could increase confidence between parents or legal guardians and adolescents, improving health behaviors and including PA in the routine and lifestyle of adolescents. Public policies should encourage the practice of PA in the family context, especially during adolescence.
